# The Use of Tobacco

**Published:** 1889-01

**Authors:** 


### THE USE OF TOBACCO.

### WHERE THE VIRTUES AND VICES OF THE WEED LIE—SMOKERS AND NON-SMOKERS.

Tobacco contains an acrid, dark-brown oil, an alkaloid, nicotine, and another substance called nicotianine, in which exist its odorous and volatile principles. When tobacco is burned, a new set of substances is produced, some of which are less harmful than the nicotine, and are more agreeable in effect, and much of the acrid oil–a substance quite as irritating and poisonous as nicotine—is carried off. These fire-produced substances are called, from their origin, the "pyridine series." By great heat the aromatic and less harmful members of the series are produced, but the more poisonous compounds are generated by the slow combustion of damp tobacco. This oil which is liberated by combustion is bad both in flavor and in effect, and it is better, even for the immediate pleasure of the smoker, that it should be excluded altogether from his mouth and air passages.

Smoking in a stub of a pipe is particularly injurious, for the reason that in it the oil is stored in a condensed form, and the smoke therefore highly charged with the oil. Sucking or chewing the stub of a cigar that one is smoking is a serious mistake, because the nicotine in the unburned tobacco dissolves freely in the saliva, and is absorbed. "Chewing" is on this account the most injurious form of the tobacco habit, and the use of a cigar holder is an improvement on the custom of holding the cigar between the teeth. Cigarrettes are responsible for a great amount of mischief, not because the smoke from the paper has any particularly evil effect, but because smokers—and they are often boys or very young men—are apt to use them continuously or at frequent intervals, believing that their power for evil is insignificant. Thus the nerves are under the constant influence of the drug, and much injury to the system results. Moreover, the cigarette smoker uses a very considerable amount of tobacco during the course of a day. "Dipping" and "snuffing" are semi-barbarities which need not be discussed. Not much effect is obtained from the use of the drug in these varieties of the habit.

Nicotine is one of the most powerful of the "nerve poisons" known. Its virulence is compared to that of prussic acid. If birds be made to inhale its vapor in amounts too small to be measured, they are almost instantly killed. It seems to destroy life, not by attacking a few, but of all the functions essential to it, beginning at the centre, the heart. A significant indication of this is that there is no substance known which can counteract its effects; the system either succumbs or survives. Its depressing action on the heart is by far the most noticeable and noteworthy symptom of nicotine poisoning. The frequent existence of what is known as "smoker's heart" in men whose health is in no other respect disturbed is due to this fact.

Those who can use tobacco without immediate injury will have all the pleasant effects reversed, and will suffer from the symptoms of poisoning if they exceed the limits of tolerance. These symptoms are: 1. The heart's action becomes more rapid when tobacco is used; 2. Palpitation pain, or unusual sensations in the heart; 3. There is no appetite in the morning, the tongue is coated, delicate flavors are not appreciated, and acid dyspepsia occurs afer eating; 4. Soreness of the mouth and throat, or nasal catarrh appears, and becomes very troublesome; 5. The eyesight becomes poor, but improves when the habit is abandoned; 6. A desire, often a craving for liquor or some other stimulant, is experienced.

In an experimental observation of thirty-eight boys of all classes of society, and of average health, who had been using tobacco for periods ranging from two months to two years, twenty-seven showed severe injury to the constitution and insufficient growth; thirty-two showed the existence of irregularity of the heart's action, disordered stomachs, cough, and a craving for alcohol; thirteen had intermittency of the pulse: and one had consumption. After they had abandoned the use of tobacco, within six months, one-half were free from all their former symtoms, and the remainder had recovered by the end of the year.

A great majority of men go far beyond what may be called the temperate use of tobacco, and evidences of injury are easily found. It is only necessary to have some record of what the general health was previous to the taking up of the habit, and to have observation cover a long enough time. The history of tobacco in the island of New Zealand furnishes a quite suggestive illustration for our purpose, and one on a large scale. When Europeans first visited New Zealand they found in the native Maoris the most finely developed and powerful men of any of the tribes inhabiting the islands of the Pacific. Since the introduction of tobacco, for which the Maoris developed a passionate liking, they have from this cause alone, it is said, become decimated in numbers, and at the same time reduced in stature and in physical well-being so as to be an altogether inferior type of men.—*New York Medical Journal.*

